# High fitness areas drive the aggregation of the sea urchin *Mesocentrotus nudus*

**DOI:** 10.7717/peerj.12820

**Published:** 2022-01-19

**Authors:** Yushi Yu, Jiangnan Sun, Yaqing Chang, Chong Zhao

**Affiliations:** 1 Dalian Ocean University, Key Laboratory of Mariculture & Stock Enhancement in North China’s Sea, Ministry of Agriculture and Rural Affairs, Dalian, Liaoning, China; 2 Southern Marine Science and Engineering Guangdong Laboratory, Guangzhou, China

**Keywords:** *Mesocentrotus nudus*, Aggregation, Fitness, Kelp forests

## Abstract

Sea urchin aggregation is a common phenomenon in coastlines. However, it remains controversial whether sea urchins form resource aggregations or behavioral aggregations in a non-spawning season. To clarify, we studied the aggregative responses to food and predators in the sea urchin *Mesocentrotus nudus* when high fitness areas (HFAs) were scarce versus sufficient. By taking the occupied area of each sea urchin (test diameter + spines =  4.5 cm) as a square (4.5 cm × 4.5 cm), we set scarce HFAs for the sea urchins in Experiment 1 (the squares of HFAs: the area occupied by experimental sea urchins = 1:1) and sufficient HFAs for the sea urchins in Experiment 2 (the squares of HFAs: the area occupied by experimental sea urchins = 2:1). If *M. nudus* form resource aggregations, they would aggregate passively under the scarce HFAs conditions, but not in the sufficient HFAs conditions. Conversely, if *M. nudus* form behavioral aggregation, aggregation would occur in both scarce and sufficient HFAs. The present results showed that in the scarce HFAs, *M. nudus* in the food and predator groups were significantly closer to the food and further from predators, and had significantly more aggregated numbers in HFAs than those in the control group. Sea urchins did not aggregate in response to food or predators under the sufficient HFAs, although significantly more sea urchins of the experimental group was found in HFAs than that of the control group. Sea urchins (at least *M. nudus*) form resource aggregations that are driven by the scarce HFAs. This provides valuable information into the mechanisms of the aggregation of sea urchins.

## Introduction

Aggregation of sea urchins is commonly known the presence of three or more individuals in cohesive groups (Vadas et al., 1986; Alvarado, 2008). Aggregations and subsequent overgrazing by sea urchins greatly accelerate the decline of kelp forests in the world (Lawrence, 1975; Ling et al., 2015; Kriegisch et al., 2019). Sea urchins aggregate in the reproductive season to increase fertilization success (Levitan, Sewell & Chia, 1992; Wahle & Peckham, 1999). However, the cause of consistently common aggregation of sea urchins in non-spawning seasons is controversial (Beddingfield, 1997; Lauzon-Guay & Scheibling, 2007).

The positive and negative stimuli point to different categories of sea urchin aggregation. Sea urchins aggregate in patches of the greatest food availability (Lauzon-Guay & Scheibling, 2007), suggesting the aggregation of sea urchins is a response to better resources that increase fitness (Scheibling, Hennigar & Balch, 1999). Pearse & Arch (1969), however, hypothesized that aggregation of the sea urchins was a defense against predators in some situations. The distinction may be caused by the complexity of the ocean. Whether sea urchins form resource (driven by resources) or behavioral aggregation (driven by social interaction) remains mostly unknown (Reese, 1966; Alvarado, 2008). It is essential to investigate the causes of aggregation of sea urchins in a non-spawning season under highly controlled laboratory conditions. Food and predators, as two typical external stimuli (Klinger & Lawrence, 1985; Spyksma, Taylor & Shears, 2017), have great effects on the aggregations of sea urchins (Vadas et al., 1986; Lauzon-Guay & Scheibling, 2007; Nichols, Segui & Hovel, 2015). The area nearest to the food or farthest from the predators are the higher fitness areas (HFAs) because the addition of food directly creates higher fitness for sea urchins in the vicinity and the addition of predators creates lower fitness for sea urchins in the vicinity (Lemire & Himmelman, 1996). If sea urchins form resource aggregation, the sea urchins would be attracted to HFAs by resources that results in higher fitness. Thus, sea urchins would passively aggregate under scarce HFAs condition. Alternatively, sea urchins would actively aggregate for social interactions in both scarce and sufficient HFAs, if they form behavioral aggregations.

The sea urchin *Mesocentrotus nudus*, is found in in kelp forests of northeast Asian seas (Zhen, 2016; Agatsuma et al., 2019). Overgrazing by *M. nudus* causes deterioration of kelp beds (Agatsuma et al., 2019). Therefore, it is a representative species and an ideal model to study the phenomenon of sea urchin aggregation (Agatsuma et al., 2019; Agatsuma, 2020). The brown alga *Saccharina japonica* and the crab *Charybdis japonica*, as natural food and a common predator of *M. nudus*, are ideal cues for testing the causes of aggregation of *M. nudus* (Zhen, 2016; Agatsuma et al., 2019)*.* The main purpose of the present study is to investigate the potential causes of the aggregation of *M. nudus* (resource-driven or behavioral-driven) in the non-spawning season and to provide valuable information into the mechanisms of aggregation of sea urchins.

## Materials and Methods

### Sea urchins

Five hundred and forty *M. nudus* (45.27 ± 2.48 mm test diameter and spines) were transported from a local aquafarm to the Key Laboratory of Mariculture & Stock Enhancement in the North China’ s Sea, Ministry of Agriculture and Rural Affairs, Dalian Ocean University. Twelve of the 540 sea urchins were randomly selected and dissected before the experiments. No sexual maturation or spawning was found in the samples (*N* = 12). Then, the rest 528 sea urchins were temporarily cultured with aerated seawater at ambient temperature (20 ± 1.0 °C) for 15 days in a tank (length × width × height: 85  × 50  × 65 cm, ∼276 L) under the natural photoperiod (12L: 12D) for the aggregation experiments. They were fed *S. japonica ad libitum* every three days. Sea urchins was subsequently held without food for three days before the experiment started on July 26, 2019. The filtered natural seawater was changed every three days. The 528 sea urchins were used in two experiments, including 352 urchins for Experiment 1 (224 in the food test, 128 in the predator test) and another 176 urchins for Experiment 2 (112 in the food test, 64 in the predator test). No sea urchin was used more than once in the experiments.

### Experiment 1

**Figure 1:**
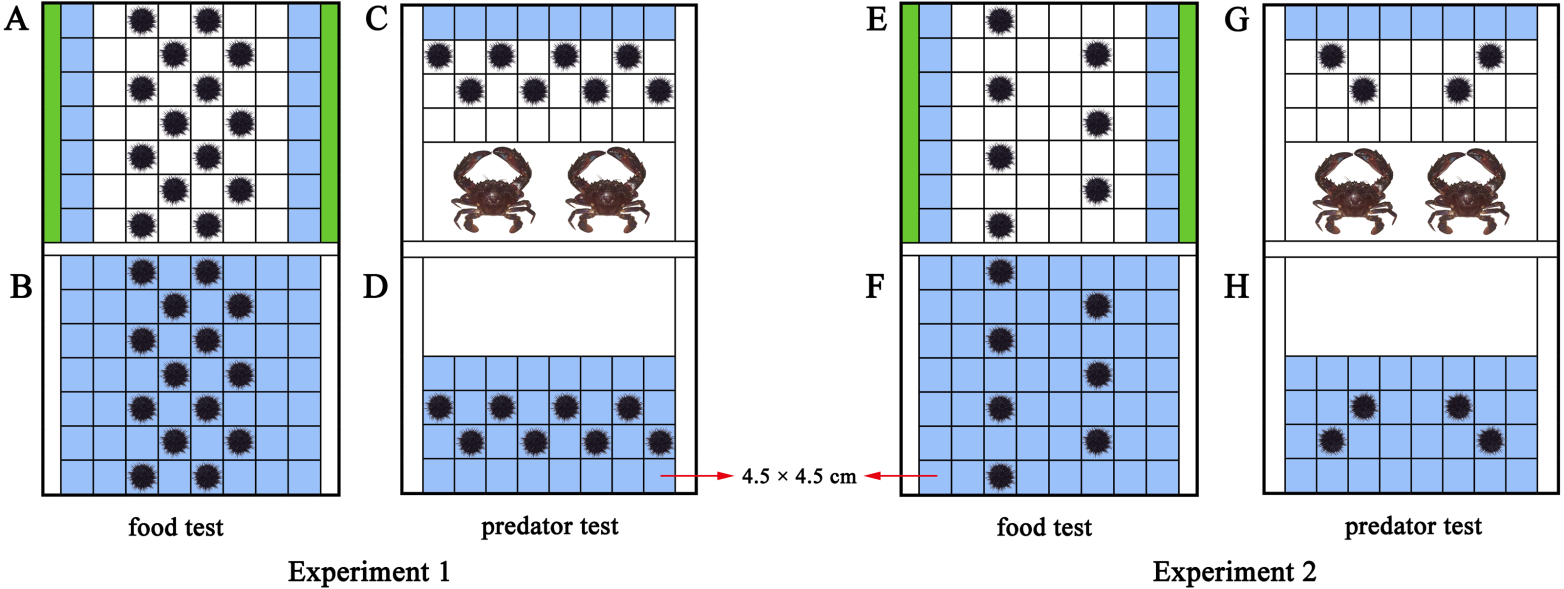
Diagram of the experimental set-ups. The blue squares were the higher fitness areas (HFAs) and the green contained kelp. The upper part is the experimental set-up of the food and predator groups (A, C, E, G), the lower part is the experimental set-up for the control groups (B, D, F, H). The nets excluded sea urchins from the white areas in D and H.

An acrylic rectangular tank divide into two parts (length × width × height: 42  × 31.5 × 17.5 cm of each part) was used to investigate how *M. nudus* aggregate in response to food. Fixed polyvinyl nets at 3 cm from both sides of the tank separated the tank into a food part and a sea urchin part (Figs. 1A, S1). We took the occupied area of each sea urchin (test diameter + spines = ∼4.5 cm) as a square (4.5 cm × 4.5 cm). Therefore, 56 squares (4.5 cm × 4.5 cm) were marked in the experimental set-up for sea urchins (8  × 7 squares) as the total area for sea urchins. Pieces of *S. japonica* (15 g) were placed in both sides of the tank for the food group, making the 14 squares nearest to the kelp the HFAs. Fourteen sea urchins were subsequently placed on the squares of the tank. No food and special HFAs was involved in the control group with 14 sea urchins (Figs. 1B, S1). Twenty-eight sea urchins were used in each replicate of the food text (14 for the food group, 14 for the control group). The food test was repeated eight times in darkness with different sea urchins and kelp (*N* = 8).

To further investigate whether aggregation of sea urchins depends on various resources or is only induced by food, we performed a test of negative stimulus using the predator *C. japonica*. Similar to the food tank, the experimental set-up was an equally divided identical tank (length × width × height: 42  × 31.5 × 17.5 cm of each part) with additional fixed nets at 18 cm from the short side to separate predators from sea urchins (Figs. 1C, S2). Thirty-two squares were marked as the place to put in sea urchins. Two *C. japonica* were placed in the other side of the nets after one-week without food, making the eight squares farthest from the crabs as the HFAs for the eight *M. nudus* of the predator group. The fitness of the eight *M. nudus* in the control group without predator was the same in all squares (Figs. 1D, S2). Sixteen sea urchins were used in each replicate of the predator test (8 for the predator group, 8 for the control group). The predator test was repeated eight times in darkness using different sea urchins and kelp/crabs (*N* = 8).

Sea urchins were placed in diagonal squares (without contacting each other) at the beginning of the experiment and then allowed to move (Fig. 1). The behaviors were recorded for 30 min using an infrared digital camera (Legria HF20; Canon, Tokyo, Japan). We changed the seawater and washed the experimental tanks for each trial.

### Experiment 2

The HFAs were relatively scarce for *M. nudus* in Experiment 1 (the squares of HFAs: the area occupied by experimental sea urchins = 1:1), because sea urchins would inevitably aggregate after they all migrated to HFAs at the ratio of 1:1. Alternatively, the ratio of 2:1 was set as a sufficient HFAs condition (the squares of HFAs: the area occupied by experimental sea urchins = 2:1). Because sea urchins have sufficient space of HFAs, they would remain separated from each other even when they all moved to the HFAs. Experiment 2 was thus designed to observe the aggregation of sea urchins under the sufficient HFAs.

The same experimental set-up in Experiment 1 was used in Experiment 2. With the addition of 15 g kelp for both sides of the experimental set-up of the food group, the 14 squares remain the HFAs for the *M. nudus* (Fig. 1E). But the number of sea urchins was halved to seven to set the ratio 2:1 (14 squares of HFAs for seven sea urchins). There was no special HFAs for the seven *M. nudus* in the control group, because no food was involved (Fig. 1F). Fourteen sea urchins were used in each replicate (seven for the experimental group, 7 for the control group). The food test was repeated eight times with different sea urchins and kelp (*N* = 8).

Likewise, two crabs were placed for the predator group after the sea urchins were not fed for a week, resulting in eight squares of the HFAs. To create sufficient HFAs condition for sea urchins, we subsequently put four *M. nudus* in the tank (Fig. 1G). The four *M. nudus* were not exposed to predators in the control group with the same fitness in all squares (Fig. 1H). The predator test was repeated 8 times using different sea urchins and crabs (*N* = 8). Eight sea urchins were used in each replicate (four for the experimental group, four for the control group).

### Statistical analysis

We calculated the total number of squares between sea urchins and the nets, the number of sea urchins in the HFAs and the number of aggregated sea urchins in groups according to the 30 min video recordings. The total number of squares between sea urchins and nets was the sum of the minimum distance (measured in squares) between each sea urchin and the net. Aggregation was considered as the grouping of two or more sea urchins, according to the definition of aggregation (Vadas et al., 1986). The corresponding squares to the HFAs in the food/predator group were used as the HFAs in the control group.

The data were analyzed for homogeneity of variance and normal distribution using Levene’s test and Kolmogorov–Smirnov test before statistical analysis, respectively. All data showed normal distribution and homogeneity of variance in Experiment 1, while some of the data in Experiment 2 did not show normal distribution and/or homogeneity of variance. In Experiment 1, therefore, independent sample t-tests were performed to detect differences in the number of squares between sea urchins and the nets, the number of aggregated sea urchins and the number of sea urchins in HFAs. In Experiment 2, independent sample t-tests were performed to detect differences in the number of squares between sea urchins and the nets, the number of aggregated sea urchins in food test. The nonparametric Mann–Whitney test was used to analyze the number of sea urchins in HFAs in the food test, the number of aggregated sea urchins and the number of sea urchins in HFAs in the predator test. All statistical analyses were performed using SPSS 25.0 statistical software. A probability level of *P* <0.05 was considered as being significant.

## Results

### Experiment 1

**Figure 2:**
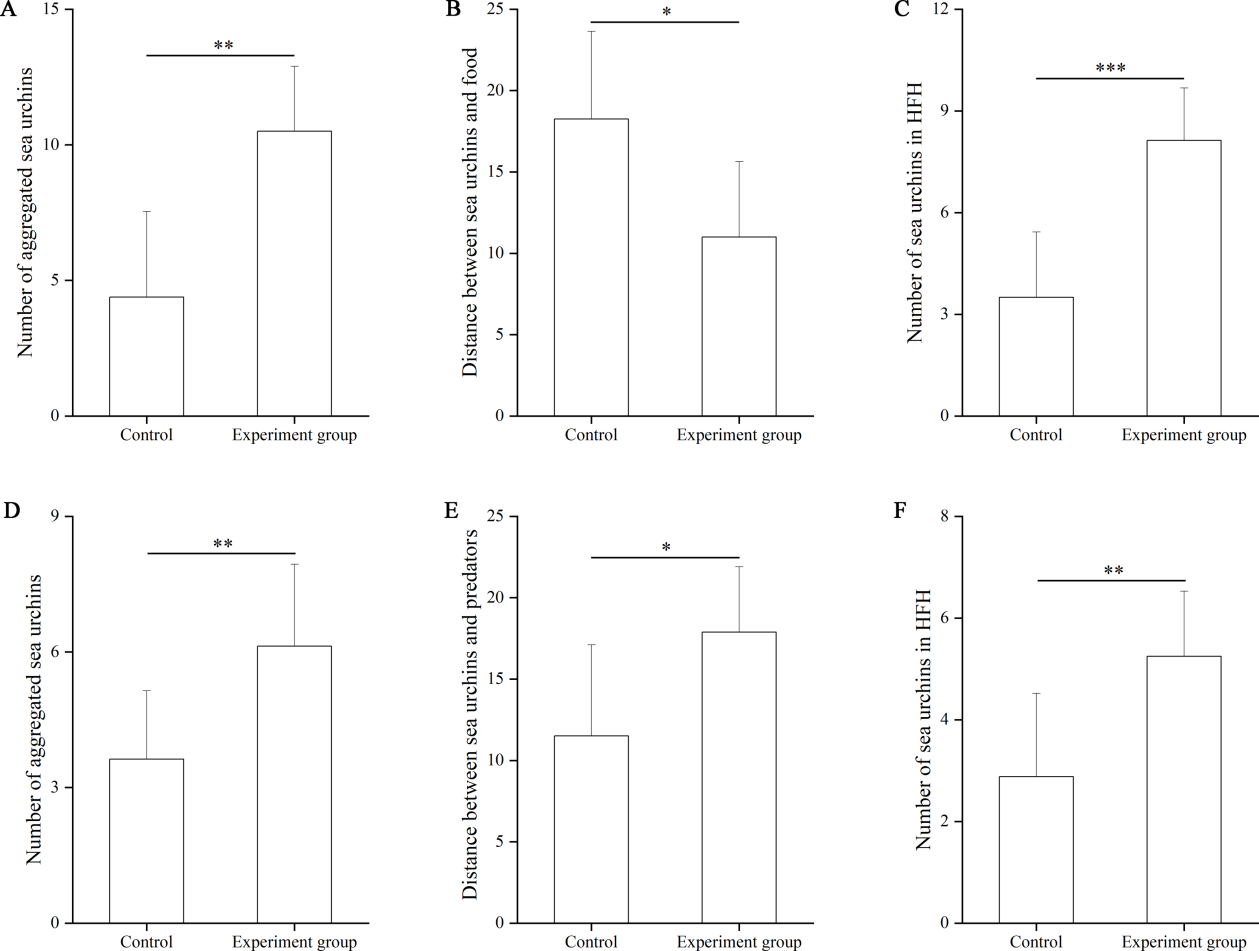
The number of aggregated sea urchins, the number of squares between sea urchins and nets, the number of sea urchins in higher fitness areas (HFAs) in the food (A, B, C) and the predator (D, E, F) tests in Experiment 1 (*N* = 8, mean ± SD). Significant differences are marked as ^*^, ^**^, ^***^ for *P* < 0.05, *P* < 0.01, *P* < 0.001, respectively.

In the food test, the number of aggregated sea urchins was significantly larger in the food group (10.50 ± 2.39) than in the control group (4.38 ± 3.16, *df* = 7, *F* = 0.962, *P* = 0.009, Fig. 2A). The number of squares between sea urchins and the nets was significantly smaller in the experimental group (11.00 ± 4.63 squares) than that in the control group (18.25 ± 5.38, *df* = 7, *F* = 0.024, *P* = 0.012, Fig. 2B). The number of sea urchins in the HFAs of the food group (8.13 ± 1.56) was significantly larger than that of the control group (3.50 ± 1.93, *df* = 7, *F* = 0.057, *P* < 0.001, Fig. 2C).

In the predator test, the number of aggregated sea urchins of the predator group (6.13 ± 1.81) was significantly larger than that of the control group (3.88 ± 1.51, *df* = 7, *F* = 0.047, *P* = 0.009, Fig. 2D). The number of squares between sea urchins and the nets was significantly larger in the predator group (17.88 ± 4.02) than that in the control group (11.50 ± 5.61, *df* = 7, *F* = 1.757, *P* = 0.020, Fig. 2E). The number of sea urchins in the HFAs was significantly larger in the predator group (5.25 ± 1.28) than those in the control group (2.88 ± 1.64, *df* = 7, *P* = 0.006, Fig. 2F).

### Experiment 2

**Figure 3:**
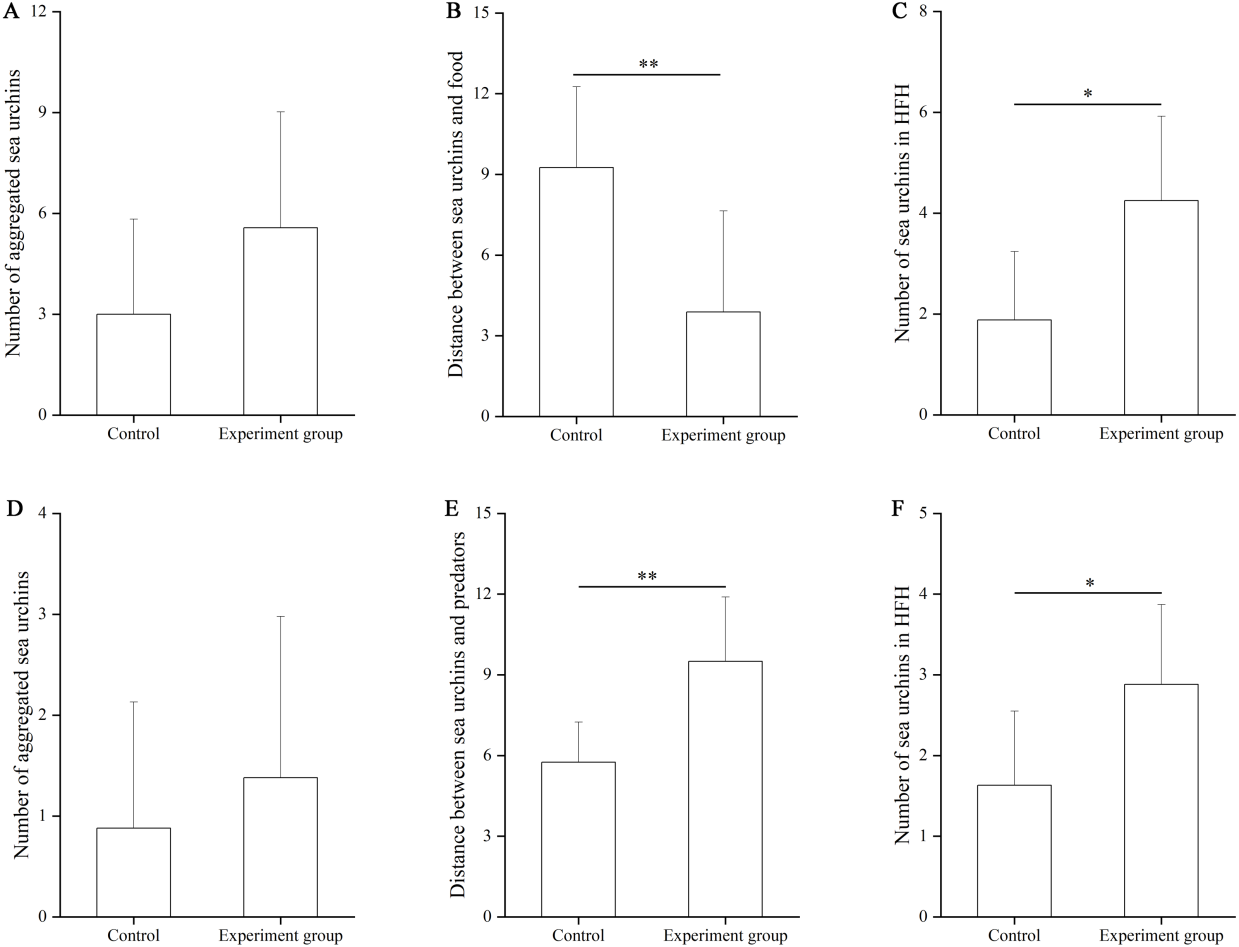
The number of aggregated sea urchins, the number of squares between sea urchins and nets, the number of sea urchins in higher fitness areas (HFAs) in the food (A, B, C) and the predator (D, E, F) tests in Experiment 2 (*N* = 8, mean ± SD). Significant differences are marked as ^*^, ^**^ for *P* < 0.05, *P* < 0.01, respectively.

In the food test, no significant difference was found in the number of aggregated sea urchins between the control and the food groups (*df* = 7, *F* = 0.364, *P* = 0.103, Fig. 3A). The number of squares between sea urchins and the nets was significantly larger in the control group (9.25 ± 3.01 squares) than that in the food group (3.88 ± 3.76 squares, *df* = 7, *F* = 0.559, *P* = 0.007, Fig. 3B). The number of sea urchins in the HFAs was significantly larger in the food group (4.25 ± 1.67) than that in the control group (1.88 ± 1.36, *df* = 7, *U* = 8.50, *P* = 0.010, Fig. 3C).

In the predator test, no significant difference of aggregation was found between the control and the predator group (*df* = 7, *U* = 26.50, *P* = 0.574, Fig. 3D). The number of squares between sea urchins and the nets was significantly bigger in the predator group (9.50 ± 2.39) than that in the control group (5.75 ± 1.49, *df* = 7, *F* = 3.402, *P* = 0.002, Fig. 3E). The number of sea urchins in the HFAs was significantly larger in the predator group (2.88 ± 0.99) than in the control group (1.63 ± 0.92, *df* = 7, *U* = 12.50, *P* = 0.038, Fig. 3F).

## Discussion

We found that *M. nudus* tend to occupy HFAs and aggregate to food, supporting the higher fitness value in the vicinity of food (Vadas et al., 1986; Lemire & Himmelman, 1996; Scheibling, Hennigar & Balch, 1999). This suggests that the aggregation is a response to the attraction of food that would increase fitness (Lauzon-Guay & Scheibling, 2007; Vadas et al., 1986). To further investigate whether the aggregation is triggered by HFAs or just by food, we observed how sea urchins responded to the exposure of predators. Similarly, no aggregation of sea urchins was found in the control group, since no predator and no special HFAs was involved. Unsurprisingly, sea urchins of the predator group moved to the HFAs, which were further from the predators. This suggests that the positive cues (for example, food) and negative cues (for example, predators) both affect the fitness of the area. The HFAs is probably the driver of aggregation, because sea urchins tend to live in areas of high fitness. The present results showed that sea urchins aggregated to stimuli under scarce HFAs conditions (the squares of HFAs: the area occupied by experimental sea urchins = 1:1), further supporting that the aggregation of sea urchins is probably driven by HFAs. This suggests that the aggregation of sea urchins is a resource type in non-spawning seasons, although the behavioral aggregation is not excluded.

To further reveal the category of the aggregation (resource or behavioral aggregation), we investigated the response of *M. nudus* under sufficient HFAs condition (the squares of HFAs: the area occupied by sea urchins = 2:1). The sea urchins retained the tendency of avoiding predators and approaching food as the strategies of pursuing higher fitness. However, no significant aggregation was found in these higher fitness-pursuing urchins. The aggregation only occurs when sea urchins are exposed to the scarce HFAs, rather than in exposure to sufficient HFAs. This indicates that the stimuli-induced aggregations are essentially responses to the HFAs. Sea urchins are driven to HFAs to increase fitness and are thus forced to aggregate when the HFAs are scarce. These results suggest that *M. nudus* form a resource aggregation, rather than a behavioral aggregation.

Resource aggregation commonly exists in non-social animals, including the locust *Maladera matrida* and the bed bug *Cimex lectularius L.* (Harari, Benyaki & Rosen, 1994; Hentley et al., 2017). This supports the opinion that the aggregation of sea urchins is an emergent pattern resulting from individuals rather than a social aggregation (Parrish & Edelstein-Keshet, 1999; Abraham, 2007). Aggregation is considered as a positive defensive behavior of sea urchins against predators (Dayton, 1971; Warner, 1979; Bernstein, Schroeter & Mann, 1983; Bernstein, Kacelnik & Krebs, 1988). However, the present study indicates that the protective function is probably a consequence, rather than a cause. Consistently, functions (for example, producing phenylacetonitrile against predators) occur after the aggregation of the locust *Locusta migratoria migratorioides* (Torto et al., 1994; Abdelatti & Hartbauer, 2020). In addition, the present results well explain the finding of Bernstein, Schroeter & Mann (1983) that predators induce the hiding of sea urchin at low density while the aggregation at high density. This is probably because that HFAs are sufficient for sea urchins at low density, but scarce at high density.

Further, it remains debatable in the high biomass of sea urchins in barrens, where they cannot obtain sufficient food (Filbee-Dexter & Scheibling, 2014; Ling et al., 2019). Although sea urchins aggregate for reproduction benefits (Wahle & Peckham, 1999; McCarthy & Young, 2004), aggregation in barrens can be found in juvenile sea urchins or in non-spawning seasons (Vadas et al., 1986; Gagnon, Himmelman & Johnson, 2004). This phenomenon can be well explained by the present finding of HFAs that the barrens may benefit sea urchins due to the lack of predators (Ling et al., 2019). Consistently, the barrens are mostly occupied by small sea urchins that have weaker defenses in exposure to predators and lower ingestion rate than the large individuals (Vadas, 1986; (Gagnon, Himmelman & Johnson, 2004). Notably, cultured sea urchins differ from wild sea urchins in behaviors (Unuma et al., 1996; Hori & Noda, 2007). The complex environments probably increase the differences in field (McCarthy & Young, 2014; Zhadan & Vaschenko, 2019). The present study is a laboratory investigation using farmed sea urchins without considering the complexity of the field. Future investigation is thus essential to test whether sea urchins form the resource aggregation in the field.

## Conclusions

The aggregation of *M. nudus* induced by positive cues (for example, food) and negative cues (for example, predators) is essentially responding to the HFAs. This indicates that *M. nudus* form a resource aggregation in the non-spawning season, which is passively regulated by the scarce HFAs but not by the sufficient HFAs. The present results provide valuable information into the mechanisms of aggregation behavior of sea urchins and highlight the importance of establishing HFAs to regulate the aggregation of sea urchins.

## Supplemental Information

10.7717/peerj.12820/supp-1 Supplemental Information 1 Raw data

10.7717/peerj.12820/supp-2 Supplemental Information 2 Diagram of the food experimental set-ups The upper part is the experimental set-up of the food groups (A for experiemnt 1, C for experiment 2), the lower part is the experimental set-up for the control groups (B for experiemnt 1, D for experiment 2).

10.7717/peerj.12820/supp-3 Supplemental Information 3 Diagram of the predator experimental set-ups The upper part is the experimental set-up of the predator groups (A for experiemnt 1, C for experiment 2), the lower part is the experimental set-up for the control groups (B for experiemnt 1, D for experiment 2).

## Additional Information and Declarations

### Data Availability

The following information was supplied regarding data availability: The raw measurements are available in the Supplementary File.
