# Supplementary figures and images for: High fitness areas drive the aggregation of the sea urchin Mesocentrotus nudus
Source: PeerJ. 2022 Jan 19;10:e12820. doi: 10.7717/peerj.12820 (PMC8783556; doi:10.7717/peerj.12820)

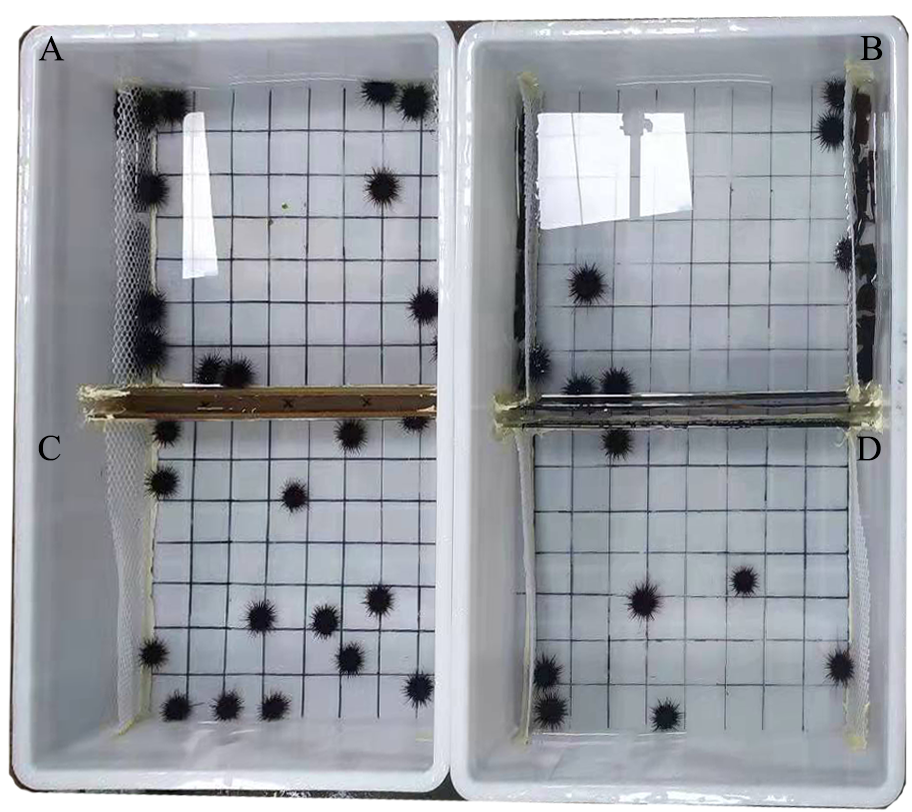

Supplement: Supplemental Information 2 — The upper part is the experimental set-up of the food groups (A for experiemnt 1, C for experiment 2), the lower part is the experimental set-up for the control groups (B for experiemnt 1, D for experiment 2). [file peerj-10-12820-s002.png]

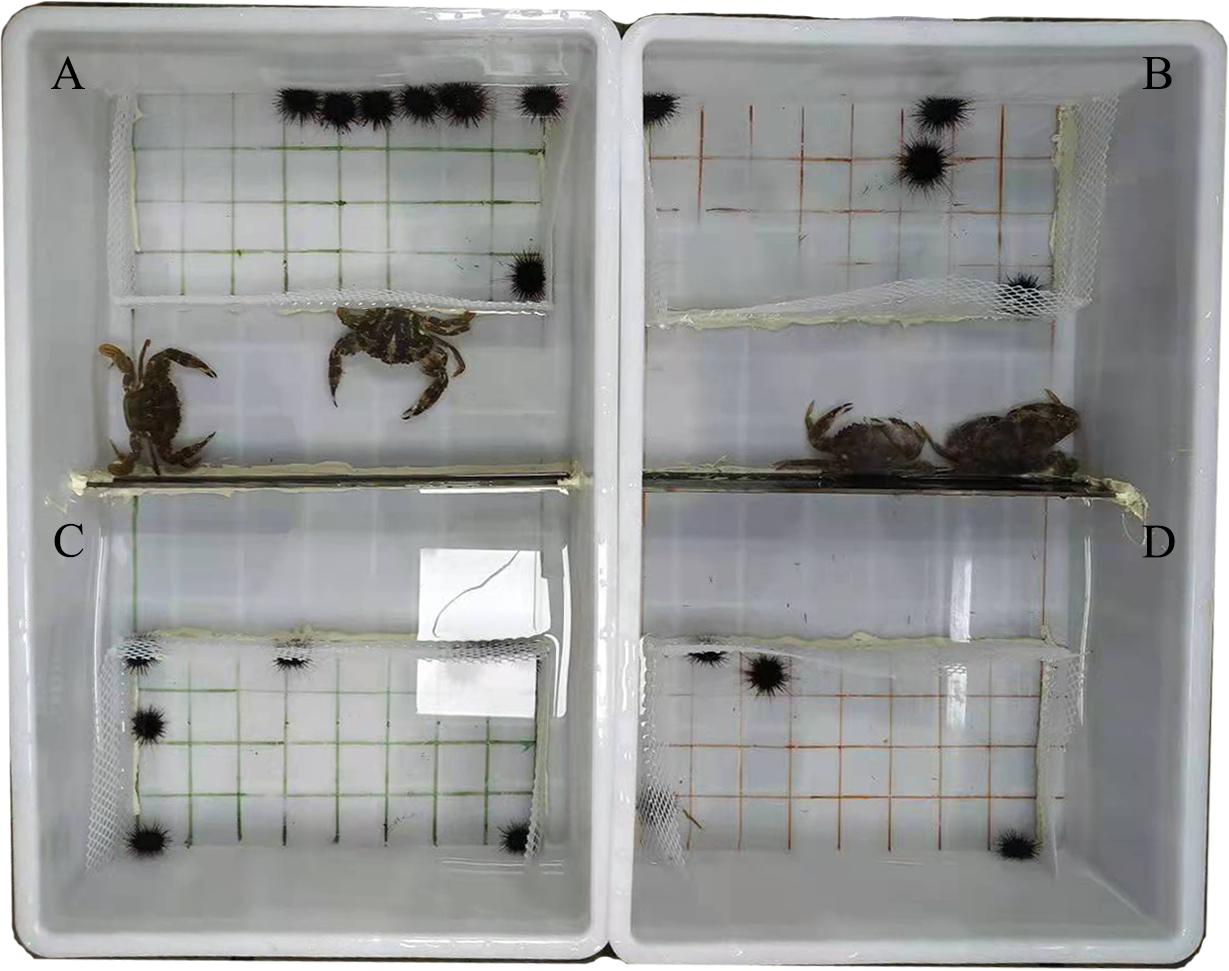

Supplement: Supplemental Information 3 — The upper part is the experimental set-up of the predator groups (A for experiemnt 1, C for experiment 2), the lower part is the experimental set-up for the control groups (B for experiemnt 1, D for experiment 2). [file peerj-10-12820-s003.png]
